# Novel Role of JAC1 in Influencing Photosynthesis, Stomatal Conductance, and Photooxidative Stress Signalling Pathway in *Arabidopsis thaliana*

**DOI:** 10.3389/fpls.2020.01124

**Published:** 2020-07-29

**Authors:** Weronika Czarnocka, Anna Rusaczonek, Patrick Willems, Marzena Sujkowska-Rybkowska, Frank Van Breusegem, Stanisław Karpiński

**Affiliations:** ^1^ Department of Botany, Institute of Biology, Warsaw University of Life Sciences, Warsaw, Poland; ^2^ Department of Plant Genetics, Breeding and Biotechnology, Institute of Biology, Warsaw University of Life Sciences, Warsaw, Poland; ^3^ Department of Plant Biotechnology and Bioinformatics, Ghent University, Ghent, Belgium; ^4^ Center of Plant Systems Biology, VIB, Ghent, Belgium

**Keywords:** chloroplast movement, stomata, photosynthesis, oxidative stress, transcriptome

## Abstract

Regulation of light absorption under variable light conditions is essential to optimize photosynthetic and acclimatory processes in plants. Light energy absorbed in excess has a damaging effect on chloroplasts and can lead to cell death. Therefore, plants have evolved protective mechanisms against excess excitation energy that include chloroplast accumulation and avoidance responses. One of the proteins involved in facilitating chloroplast movements in *Arabidopsis thaliana* is the J domain-containing protein required for chloroplast accumulation response 1 (JAC1). The function of JAC1 relates to the chloroplast actin filaments appearance and disappearance. So far, the role of JAC1 was studied mainly in terms of chloroplasts photorelocation. Here, we demonstrate that the function of JAC1 is more complex, since it influences the composition of photosynthetic pigments, the efficiency of photosynthesis, and the CO_2_ uptake rate. JAC1 has positive effect on water use efficiency (WUE) by reducing stomatal aperture and water vapor conductance. Importantly, we show that the stomatal aperture regulation is genetically coupled with JAC1 activity. In addition, our data demonstrate that JAC1 is involved in the fine-tuning of H_2_O_2_ foliar levels, antioxidant enzymes activities and cell death after UV-C photooxidative stress. This work uncovers a novel function for JAC1 in affecting photosynthesis, CO_2_ uptake, and photooxidative stress responses.

## Introduction

Plants regulate the light absorption under naturally variable conditions, which is essential for photosynthesis optimization and acclimation. Light energy absorbed in excess (excess excitation energy, EEE) leads to photoinhibition and chloroplast retrograde-signalling and may cause chloroplast damage and ultimately cell death (Mühlenbock et al., 2008; Wituszyńska and Karpiński, 2013; Wituszyńska et al., 2015; Czarnocka and Karpiński, 2018; Górecka et al., 2020). Therefore, plants have evolved EEE avoidance and dissipation mechanisms to ensure the proper functionality of the photosynthetic apparatus and photosynthesis. One of these mechanisms is chloroplast movement (Demarsy et al., 2018).

Chloroplast photorelocation is an essential mechanism to adapt to the fluctuating light conditions (Gabryś, 2004; Suetsugu et al., 2010a). In the dark, chloroplasts are located along the anticlinal walls and at the cells’ bottom. Upon transferring plants to weak or moderate light, chloroplasts reposition themselves along the periclinal walls in order to efficiently capture light energy, which is termed the accumulation response. However, after exposure to strong light intensities, chloroplasts demonstrate avoidance response, which manifests itself by positioning at anticlinal cell walls and is aimed at lowering the risk of photoinhibition and photodamage (Wada and Kong, 2018). So far, only a couple of proteins have been identified to facilitate light intensity perception and light-mediated chloroplasts movements. Well-characterized are blue and UV-A/B photoreceptors, phototropins. *Arabidopsis thaliana* (Arabidopsis) possesses two such receptors, phototropin 1 (phot1) and phototropin 2 (phot2), that redundantly mediate the chloroplast accumulation response (Sakai et al., 2001). However, the avoidance response is regulated only by phot2 and not by phot1 (Kagawa et al., 2001). In Arabidopsis, the movement of chloroplasts and their anchoring to the plasma membrane is dependent on short actin filaments located along the chloroplast periphery (cp-actin filaments). Under light induction cp-actin filaments relocalize to the leading edge of chloroplasts and cause chloroplast movement that is regulated by both phot1 and phot2 (Kadota et al., 2009), and additionally by a J-domain protein required for chloroplast accumulation response 1 (JAC1) (Ichikawa et al., 2011).

JAC1 was originally identified in an Arabidopsis mutant screen deficient in chloroplast accumulation responses (Suetsugu et al., 2005). In *jac1* mutant, chloroplasts remain localized to the side walls of palisade cells (Suetsugu et al., 2005; Gotoh et al., 2018). Apart from the important role in chloroplast accumulation at the cell surface in weak light, JAC1 is also required for the chloroplast relocation to the cell’s bottom in the darkness (Suetsugu et al., 2005). In palisade cells, the chloroplast dark positioning in the *jac1* mutant is different from wild-type plants, because chloroplasts gather at the anticlinal cell walls (Hermanowicz et al., 2019). In addition, it was proven that under excess light, JAC1, to some extent, is also needed in the regulation of the avoidance response as cp-actin filament reorganization is impaired in *jac1* (Kodama et al., 2010; Ichikawa et al., 2011). A similar perturbed cp-actin reorganization was observed in two other mutants, *weak chloroplast movement under blue light 1* (*web1*) and *plastid movement impaired 2* (*pmi2*) (Kodama et al., 2010), which show weak chloroplast movement during accumulation and avoidance responses. Even though the physical interaction between phot1 and phot2 (Sztatelman et al., 2016), as well as between WEB1 and PMI2 has been documented (Kodama et al., 2010), JAC1 does not physically interact with any of them (Suetsugu et al., 2005; Kodama et al., 2010). Therefore, a yet unidentified signal from plasma membrane-localized phototropins is received by JAC1 and passed along to chloroplasts to regulate the direction of their movement (Suetsugu et al., 2005). Moreover, it was shown that JAC1 is dispensable for phot2-mediated chloroplast avoidance response induced by high-light irradiation (Suetsugu et al., 2005).

The Arabidopsis JAC1 protein is 651 amino acids long and contains a 70 amino acid long auxilin-like J-domain at the C-terminus, which is essential for JAC1-mediated chloroplast photorelocation (Takano et al., 2010). A sequence-based classification assigned JAC1 to the group of type III J-proteins, including cochaperones of the heat shock protein 70 (Hsp70) chaperones (Takano et al., 2010). The JAC1 J-domain is similar to the J-domain of auxilins, enzymes that uncoat clathrin from the mature vesicles to promote the recycling of clathrin during endocytosis (Suetsugu et al., 2005). The highly conserved His-Pro-Asp (HPD) tripeptide within J-domain has been shown to facilitate the interactions with Hsp70 chaperones (Jiang et al., 2003). Structural analysis of the JAC1 J-domain indicates that HPD motif is indispensable for JAC1-dependent chloroplast light-induced movement and suggests that JAC1 cooperation with heat shock protein cognate 70 (Hsc70) chaperone is needed in this process (Suetsugu et al., 2010b). Auxilin J-domain targets and activates the Hsc70 that mediates clathrin uncoating (Eisenberg and Greene, 2007). *JAC1* overexpression was shown to inhibit endocytosis process in root hair cells, which indirectly supports its role in clathrin uncoating (Ezaki et al., 2007). However, so far there is no evidence on the involvement of Hsc70 and clathrin-mediated endocytosis in chloroplast movement (Adamowski et al., 2018). The localization of JAC1 is most probably cytoplasmic, but its presence in the nucleus (Suetsugu et al., 2005) or within cellular membranes, cannot be excluded (Suetsugu et al., 2015). Furthermore, neither the expression level of *JAC1* gene nor the protein level are regulated by light or by the phototropins (Suetsugu et al., 2005).

Even though the role of JAC1 has been broadly studied in terms of chloroplast movements, so far little is known about the other molecular and physiological processes that involve JAC1. This study demonstrates that JAC1 influences photosynthetic reactions, stomatal aperture and thus water vapor conductance and CO_2_ uptake. This regulation, at least partially, involves JAC1-dependent gene expression changes. In addition, we prove that JAC1 takes part in the fine-tuning of photooxidative-stress induced accumulation of hydrogen peroxide (H_2_O_2_), regulation of antioxidant enzymes activities and cell death. Our results shed a new light on the role of JAC1 in Arabidopsis.

## Materials and Methods

### Plant Material and Growth Conditions

*Arabidopsis thaliana* *jac1-1* mutant in Columbia-0 gl1 (Col-0 gl1) background together with wild-type Col-0 gl1 plants were used in this study. The seeds were a kind gift from Prof. Masamitsu Wada (Tokyo Metropolitan University, Tokyo, Japan). Seeds were sown on Jiffy pots, stratified for two days in 4°C and moved for germination to standard laboratory conditions (8 h photoperiod, PPFD: 80 µmol photons m^-^² s^-^¹), 50% relative air humidity and temperature day/night: 22/18°C).

### Measurements of Morphological and Physiological Traits

All morphological and physiological parameters were measured for four-week-old plants. Rosette size was analyzed with a FluorCam (Photon System Instruments PSI, Brno, Czech Republic) for 15 plants *per* genotype in two independent experiments (n=15). Rosette dry weight was measured for 12 plants for each genotype (n=12) after desiccation in 105°C for three days. The determination of water use efficiency (WUE) was performed for 12 plants *per* genotype in two independent experiments (n=12) according to the previously published protocol (Wituszyńska et al., 2013b; Wituszynska and Karpiński, 2014).

### Microscopy and Image Analysis

The calculation of stomata number *per* mm² of leaf area was performed using the transparent glue imprints of the abaxial leaf side and with the use of Olympus AX70 Provis microscope (Olympus, Tokio, Japan). Three leaves, 6^th^, 7^th^ and 8^th^ were analyzed for 9 individual plants for each genotypes in two independent experiments (n=27). The number of stomata *per* mm² of leaf area was calculated from three frames of each microscopic sample. Stomatal aperture was measured with Image J program (Version 1.52e, National Institutes of Health, Bethesda, MD, USA) for 6^th^, 7^th^, and 8^th^ leaf, for 77 and 74 individual stomata in the wild type and *jac1* mutant, respectively (n=74–77). Arabidopsis mesophyll cells were assayed for intracellular chloroplast location with Leica TCS Sp5 confocal microscope (Leica Camera AG, Wetzlar, Germany), using chlorophyll autofluorescence upon argon laser (488 nm) excitation. 6^th^, 7^th^ or 8^th^ leaf was analyzed for 5 plants *per* each genotype *per* treatment in two independent experiments (n=5).

### Chlorophyll *a* Fluorescence and Gas Exchange Parameters Measurements

Chlorophyll *a* fluorescence parameters and OJIP test were measured with a FluorCam system (Photon System Instruments PSI, Brno, Czech Republic), including four super bright LED panels 130 x 130 mm, providing actinic light (300 µmol photons m^-2^ s^-1^) and saturation pulses (4,000 µmol photons m^-2^ s^-1^). Measurements were performed with standard “Quenching” protocol implemented in the FluorCam7 software for 11–21 plants *per* genotypes *per* treatment in two separate experiments (n=11–21). Prior the measurements, plants were dark-adapted for 30 min in order to determine *F*_0_ and *F*_m_ parameters. Chlorophyll fluorescence terminology has been previously described (Baker, 2008; Wituszyńska et al., 2013a). CO_2_ uptake and water vapor conductance (GH_2_O) in variable conditions of photosynthetic active radiation (PAR) ranging between 0 and 1,900 µmol m^-2^ s^-1^ were determined using Portable Gas Exchange System GFS-3000 (Heinz Walz GmbH, Effeltrich, Germany) for 8–10 plants *per* genotype in two independent experiments (n=8–10). In order to obtain the slope of the regression line for each tested genotype, all the values for CO_2_ uptake (or GH_2_O) for each tested PAR intensity were used. To compare the regression line slopes the procedure from Wonnacott and Wonnacott (1990) was used. The statistical analysis was performed in the “R” version 2.12.1 using stats packages.

### Photosynthetic Pigments Measurements

The determination of photosynthetic pigment compositions was performed according to the procedure described previously (Rusaczonek et al., 2015; Wituszyńska et al., 2015) for 12 plants *per* genotypes in two independent experiments (n=12).

### Hydrogen Peroxide Levels Determination

The hydrogen peroxide (H_2_O_2_) content was determined for nine plants *per* genotype *per* treatment from two independent experiments (n=9), as previously described (Rusaczonek et al., 2015; Wituszyńska et al., 2015).

### Antioxidant Enzyme Activity Measurements

The activities of superoxide dismutase (SOD), catalase (CAT), and ascorbate peroxidase (APX) were determined for nine plants *per* genotype *per* treatment from two independent experiments (n=9), as previously described (Rusaczonek et al., 2015; Wituszyńska et al., 2015).

### UV-C Treatment and Cell Death Analysis

UV-C radiation (100 mJ cm^-^²) was used as oxidative-stress inducing factor. UV-C radiation was applied with 500 Crosslinker (Hoefer Pharmacia Biotech, San Francisco, CA, USA), equipped with lamps emitting light in the wavelength ranging from 250 to 258 nm (type G8T5, 8W; Sankyo Denki, Hiratsuka, Japan). Cell death analysis was performed by the measurement of ion leakage for 9–12 plants *per* genotype *per* treatment in two independent experiments (n=9–12), as previously described (Rusaczonek et al., 2015; Wituszyńska et al., 2015). Cell death visualization was performed by staining five leaves *per* genotype *per* treatment with 1% (m/v) Evans blue and vacuum infiltration for 30 min (Mergemann and Sauter, 2000). After staining, the leaves were washed three times with deionized water and chlorophyll was removed by the incubation in chloral hydrate (2.5 mg/mL) for two days. The pictures of representative leaves are presented.

### RNA Extraction and cDNA Synthesis

The RNA was extracted from whole rosettes in three biological replicates, each consisting of at least four rosettes. Total RNA extraction was performed with a use of GeneMATRIX Universal RNA Purification Kit (EURX, Gdańsk, Poland). Additional step of on-column DNaseI digestion was performed. RNA concentration and purity were analyzed spectrophotometrically with Eppendorf BioSpectrometer (Eppendorf, Hamburg, Germany). The RNA integrity was tested by electrophoretic separation in 1% agarose gel and the same amount of RNA for each sample was used for reverse transcription. cDNA synthesis was performed with a High Capacity cDNA Reverse Transcription Kit (Thermo Fisher Scientific, Waltham, MA, USA).

### Relative Gene Expression Measurement by Real-Time qPCR

Real-time qPCR was performed for four-week-old non-treated and UV-C treated plants (3, 6, 12, and 24 h after UV-C exposure), using the 7500 Fast Real-Time PCR System and Power SYBR Green Master Mix (Thermo Fisher Scientific, Waltham, MA, USA). Each of three biological repeats *per* treatment was tested in three technical repeats. The following cycling programme was used in qPCR: 95°C for 10 min, followed by 40 cycles of denaturation in 95°C for 15 s and annealing/extension in 60°C for 60 s. Primers for *JAC1* (AT1G75100) were designed with Primer3 software (Primer3Plus, Free Software Foundation, Inc., Boston, MA, USA) and their sequence was as follows, forward 5’- gatggttctaatgccaaggaa -3’ and reverse 5’- tgaaggtttctgatcccgatt -3’. *YELLOW-LEAF-SPECIFIC GENE 8* (*YLS8*, AT5G08290) was used as a reference, according to the RefGenes tool incorporated in Genevestigator (Hruz et al., 2008) and amplified with the following primers: forward 5’- ttactgtttcggttgttctccattt-3’ and reverse 5’- cactgaatcatgttcgaagcaagt-3’. The specificity of each primer pair was analyzed by melting curve. The efficiency of real-time qPCR was calculated using LinRegPCR tool (Ramakers et al., 2003). Calculation of relative gene expression levels and the significance of difference between tested samples was performed using REST2009 (Pfaffl et al., 2002).

### RNA Sequencing and RNA-Seq Analysis

The RNA was extracted as described above from four-week-old non-treated and UV-C treated plants 30 min after exposure to 100 mJ cm^-^² of UV-C radiation. RNA was isolated from three biological replicates, each consisting of at least four rosettes. RNA concentration and purity were determined spectrophotometrically using the Nanodrop ND-1000 (Nanodrop Technologies) and RNA integrity was assessed using a BioAnalyzer 2100 (Agilent). An amount of 1,000 ng of total RNA *per* sample was used as input. Poly-A containing mRNA was purified from the total RNA, using poly-T oligo-attached magnetic beads and the Illumina TruSeq^®^ Stranded mRNA Sample Prep Kit (protocol version: Document # 1000000040498 v00 - October 2017). RNA was transcribed to cDNA in a reverse transcription reaction using random primers, and subsequently converted into double-stranded cDNA using DNA Polymerase I and RNAse H. The cDNA fragments were extended with a single ‘A’ base at the 3’ ends of the blunt-ended cDNA fragments, after which multiple indexing adapters were ligated introducing different barcodes for each sample. Finally, PCR was performed to enrich DNA fragments that possess adapter molecules on both ends. Sequence-libraries of each sample were equimolarly pooled and sequenced on Illumina HiSeq 4000 (Paired-end kit, 76 cycles, Dual Index, 4 lanes) at the VIB Nucleomics Core (www.nucleomics.be). Reads were aligned to the Arabidopsis genome by STAR (v2.5.2b) (Dobin et al., 2013), using the Araport11 annotation (Cheng et al., 2017). The number of reads *per* gene was quantified with the featureCounts function as implemented in the Subread package v1.6.2 (Liao et al., 2014). Only protein-coding genes quantified by at least 5 reads in at least three samples (20,013 genes) were retained for downstream differential gene expression analysis using the software package edgeR (Robinson et al., 2010) in R (v3.4.1). TMM normalization (Robinson and Oshlack, 2010) was applied using the calcNormFactors function. Variability in the data set was assessed with a MDS plot, showing clear separation according to genotype and UV-C treatment. In order to test user-defined hypotheses, a no-intercept single-factor model was defined combining genotype and treatment factors, e.g. such as *jac1*_UV. Dispersions were estimated with the estimateGLMRobustDisp function. A negative binomial regression model was used to model the overdispersed counts for each gene separately with fixed values for the dispersion parameter as outlined (McCarthy et al., 2012) and as implemented in the function glmFit using the above described model. Hypothesis testing was based on likelihood ratio tests. Contrasts of interest were the response between different genotypes under control conditions, the effect of UV stress in each genotype, and the interaction effect of UV stress and genotype. False discovery rate adjustments of the P values were performed with the method described by Benjamini and Hochberg (1995). The gene expression data were deposited in Gene Expression Omnibus (GEO; http://www.ncbi.nlm.nih.gov/geo/) under accession number GSE143762. Gene ontology enrichment analysis was performed using ThaleMine tool (v4.1.2-20200127) within the Araport portal (Krishnakumar et al., 2015). Functional analysis of differentially expressed genes was performed using the MapMan tool (Thimm et al., 2004).

## Results

### JAC1 Influences Photosynthetic Efficiency and the Level of Photosynthetic Pigments

**Figure 1:**
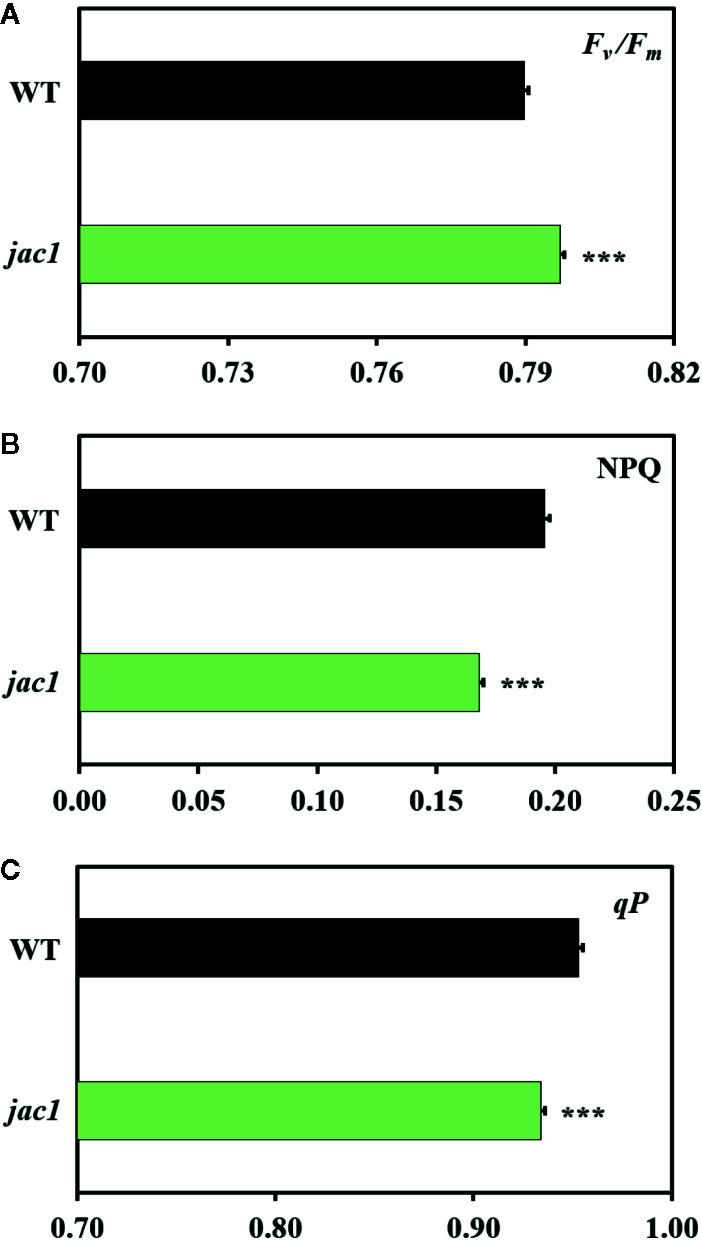
Chlorophyll *a* fluorescence parameters of 4-week-old *Arabidopsis thaliana* wild-type (WT) and *jac1* mutant. **(A)** *F_v_/F_m_*, maximum quantum efficiency of PSII; **(B)** NPQ, non-photochemical quenching; **(C)** *qP*, photochemical quenching. Values are means (± SD) of 11–21 plants *per* genotype from two independent experiments (n=11–21). Asterisks indicate significant difference in comparison with the wild-type plants at the level P < 0.001 (***) according to the Tukey’s HSD multiple comparison test.

Since JAC1 is involved in the regulation of chloroplast movements (**Supplementary Figure 1**), our first aim was to assess if it has an influence on photosynthetic efficiency. Chlorophyll *a* fluorescence measurements proved that the *jac1* mutant has higher maximum quantum efficiency of photosystem II (PSII) (*F_v_/F_m_*) (**Figure 1A**), but lower non-photochemical (NPQ) (**Figure 1B**) and photochemical quenching (*qP*) (**Figure 1C**), as compared to the wild-type plants.

In order to obtain better insight into the specific photosynthetic reactions that are JAC1-dependent, we analyzed the fluorescence transient using the O–J–I–P test (Strasser et al., 2004). This demonstrated functional changes within light harvesting complexes (LHCs) and photosystems in *jac1* mutant (**Supplementary Figure 2**). More specifically, a decreased ABS/RC ratio was apparent. This ratio represents the total number of photons absorbed by chlorophylls of all the reaction centres (RCs) divided by the total number of active RCs and thus suggests that *jac1* mutant had more active RCs than wild-type plants. Besides measuring active RCs, the TRo/RC ratio is an estimate of maximal rate by which excitation is trapped by the RC resulting in the reduction of quinone A (Q_A_). The decreased TRo/RC ratio in *jac1* suggests that not the whole Q_A_ pool was reduced in this mutant, unlike in the wild-type. Furthermore, the *jac1* mutant showed a significantly decreased reoxidation of reduced Q_A_ *via* electron transport in active RCs (ETo/RC). Because ETo/RC is represented only by the active centres, the lower ratio in the *jac1* mutant implies again that there were more active centres than in the wild type. Finally, the effective dissipation of untrapped excitation energy from all RCs with respect to the number of active RCs (DIo/RC ratio) was also lower in *jac1* mutant, indicating a better connectivity between the structurally heterogeneous units of PSII (Force et al., 2003; Wituszyńska et al., 2013a).

**Figure 2:**
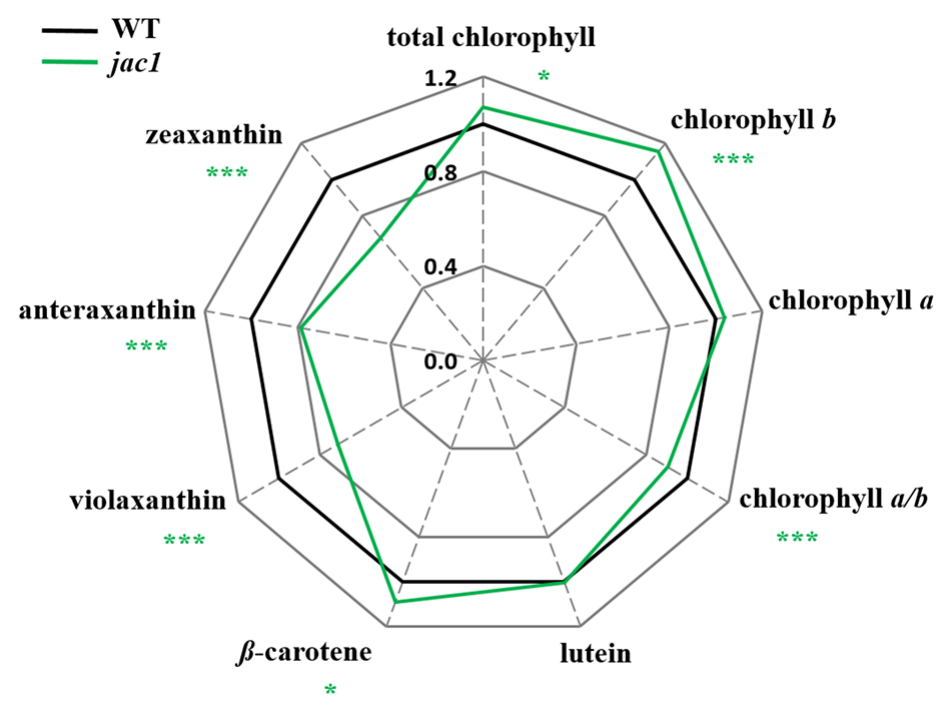
Photosynthetic pigment contents in 4-week-old *Arabidopsis thaliana* wild type and mutant plants. Total chlorophyll, chlorophyll *a*, chlorophyll *b*, chlorophyll *a/b* ratio; lutein; *β-*carotene; violaxanthin; antherxanthin; zeaxanthin. Values are means (± SD) of 12 plants *per* genotype from two independent experiments (n=12) expressed as the peak area (mg of dry weight^-1^). Asterisks indicate significant differences from the wild type according to the Tukey HSD test at the level of P < 0.05 (*), or P < 0.001 (***).

To study whether the observed JAC1-dependent changes in photosynthetic efficiency are caused by altered photosynthetic pigments levels, we analyzed their composition (**Figure 2**). The total chlorophyll level, concentration of chlorophyll *b* (but not chlorophyll *a*) were significantly elevated in *jac1*. We also observed that Chl *a/b* ratio was significantly decreased in *jac1* mutant in relation to the wild type. Moreover, while *β-*carotene level was elevated in *jac1* mutant, the content of violaxanthin, anteraxanthin and zeaxanthin were decreased in *jac1* mutant, in comparison to the control plants.

### CO_2_ Uptake Rate and Water Vapor Conductance Are Negatively Affected by JAC1

**Figure 3:**
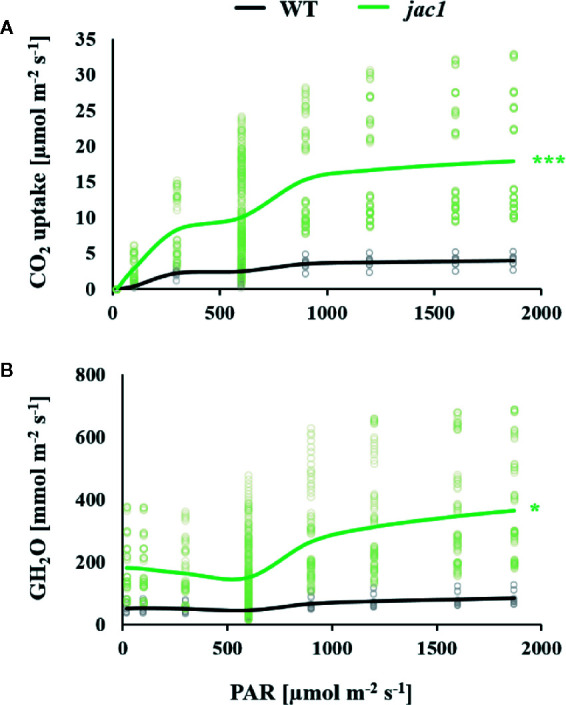
Gas exchange parameters in response to different light intensities (PAR) were measured for 4-week-old *Arabidopsis thaliana* wild type (WT) and *jac1* mutant. Graphs represent relationships between light intensity and individual photosynthetic parameters. **(A)** CO_2_ uptake and **(B)** Water vapor conductance. Values are means of 8–10 plants *per* genotype from two independent experiments (n=8–10). Asterisks indicate significant difference of regression line slopes in comparison with the wild type at the level P < 0.05 (*) or P < 0.001 (***) according to the Tukey’s HSD test.

Due to altered light-phase photosynthetic reactions in *jac1* mutant, our next step was to assess whether JAC1 influences the carbon dioxide (CO_2_) uptake. In a range of photosynthetic active radiation (PAR) intensities that were tested, the CO_2_ uptake was consistently higher in *jac1*, reaching more than three times higher uptake in high light intensities (**Figure 3A**). The simultaneously measured water vapor conductance (GH_2_O) was elevated up to three times in low PAR intensities and, similarly to the CO_2_ uptake rate, a greater relative increase in *jac1*, compared to wild-type was apparent under high PAR (**Figure 3B**). Interestingly, the variation of CO_2_ uptake and GH_2_O parameters in a single measuring point was much greater in *jac1* background, compared to the wild type.

### JAC1 Has an Impact on Water Use Efficiency Through the Reduction of Stomatal Conductance

**Figure 4:**
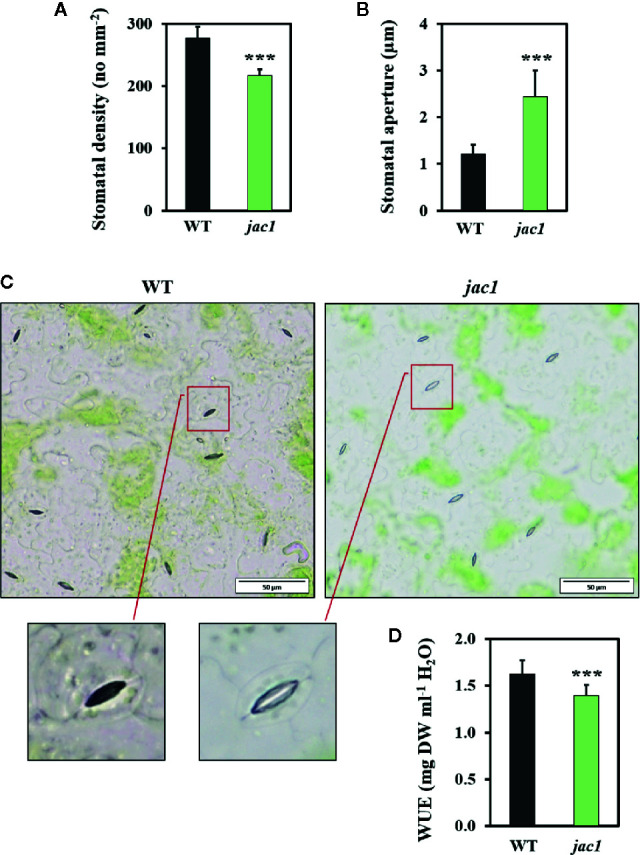
Morphological and physiological traits in 4-week-old *Arabidopsis thaliana* wild-type (WT) and *jac1* mutant. **(A)** Stomatal density. Values are means (± SD) of 27 leaf fragments *per* genotype from two independent experiments (n=27); **(B, C)** Stomatal aperture. Values are means (± SD) of 74–77 stomata *per* genotype from two independent experiments (n=74–77); and **(D)** Water use efficiency (WUE) wild type and mutant plants grown under standard laboratory conditions. Values are means (± SD) of 12 plants per genotype from two independent experiments (n=12). Asterisks indicate significant difference in comparison with the wild-type plants at the level P < 0.001 (***) according to the Tukey’s HSD test.

Such high water vapor conductance and CO_2_ uptake rate in *jac1* prompted us to analyze the impact of JAC1 activity on stomatal density and aperture. While the stomatal density was reduced in *jac1* mutant, stomatal aperture was significantly elevated (**Figures 4A–C**). Moreover, water use efficiency (WUE), measured as dry weight *per* water used, proved to be significantly decreased in *jac1* compared to wild-type plants (**Figure 4D**). Hence, *jac1* consumed relatively more water for the production of dry weight, which is in agreement with the previously observed elevated water vapor conductance (**Figure 3B**), a measure of leaf transpiration efficiency. Importantly, our results showed that the rosette size and dry weight were not different between *jac1* mutant and the wild type (**Supplementary Figure 3**, **Supplementary Figures 4A, B**).

### Photooxidative Stress Causes Changes in *JAC1* Expression Level and Shows the Influence of JAC1 on Photosynthetic Activity, Redox Homeostasis, and Cell Death Regulation

UV-C radiation has been shown to induce oxidative stress and photoinhibition. It was demonstrated that changes within chloroplasts are the first observed symptoms of UV-C induced cell death (Wituszyńska et al., 2015). Therefore, we used UV-C treatment to explore the influence of JAC1 in the photooxidative stress response and cell death

**Figure 5:**
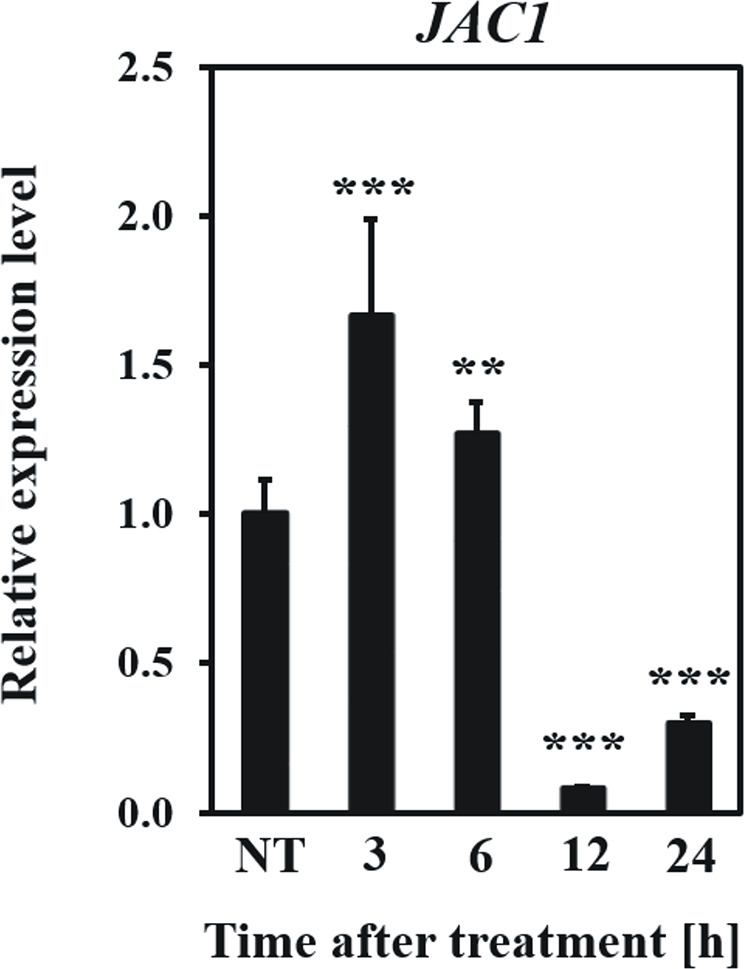
Relative expression level of *JAC1* in the *Arabidopsis thaliana* wild-type plants in non-stress conditions (NT) and 3, 6, 12, 24 h after UV-C exposure. Values are means (± SD) from three biological replicates, for which three individual qPCR reactions were performed (n=9). Asterisks indicate statistically significant differences from non-treated plants, at the level P< 0.01 (**) or P< 0.001 (***).

Firstly, we examined how the *JAC1* expression changes after UV-C exposure (**Figure 5**). In the early hours (3 and 6 h) after UV-C treatment, *JAC1* transcript abundance was slightly induced. However, between 6 and 12 h *post* UV-C stress, *JAC1* expression dropped severely to reach 10% of the initial expression level after 12 h and 30% after 24 h.

**Figure 6:**
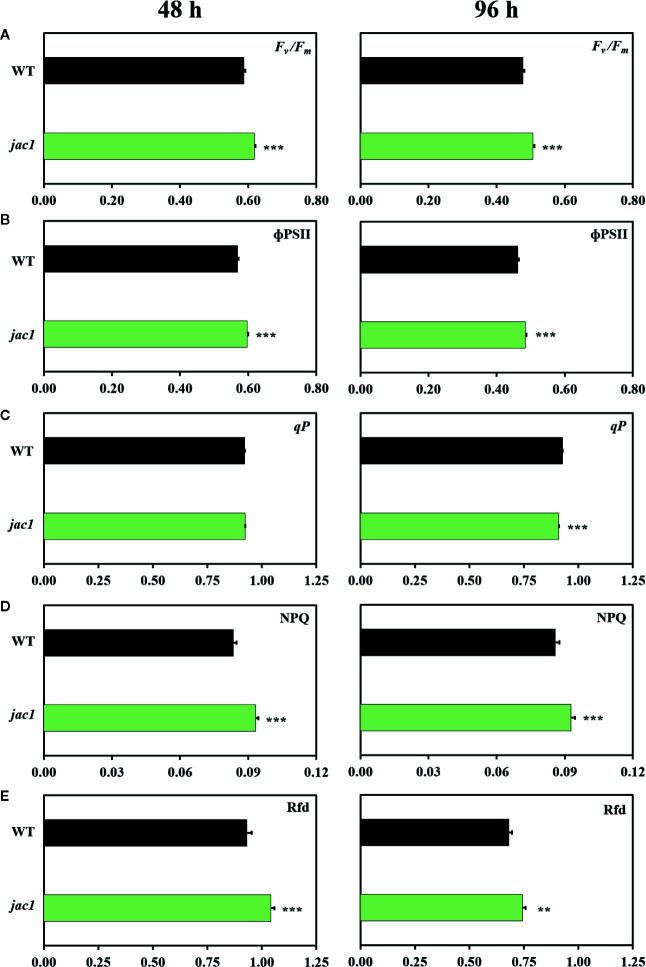
Chlorophyll *a* fluorescence parameters measured in 4-week-old *Arabidopsis thaliana* wild type and *jac1* mutant 48 and 96 h after UV-C exposure (100 mJ cm^-2^). **(A)** *F_v_*/*F_m_*, maximum quantum efficiency of PSII; **(B)** ϕPSII, quantum yield of PSII; **(C)** *qP*, photochemical quenching; **(D)** NPQ, non-photochemical quenching; **(E)** Rfd, plant vitality parameter. Values are means (± SD) of 11–21 plants *per* genotype and time point from two independent experiments (n=11–21). Asterisks indicate significant difference in comparison with the wild-type plants at the level P < 0.005 (**), or P < 0.001 (***) according to the Tukey’s HSD multiple comparison test.

In the next step, we explored the role of JAC1 during photosynthetic acclimation to oxidative stress. Our results demonstrated that both 48 and 96 h *post* UV-C exposure, *jac1* had higher values of maximum and operational quantum efficiency of PSII (*F_v_/F_m_* and ΦPSII, respectively), as compared to the wild-type plants (**Figures 6A, B**). However, 96 h after UV-C incident, *jac1* plants, compared to wild-type, exhibited decreased levels of the photochemical quenching (*qP*) parameter, signifying a lower proportion of PSII reaction centres to be open in *jac1* (**Figure 6C**). In addition, the level of EEE dissipation through the NPQ reactions was decreased in *jac1*, compared to the wild type (**Figure 6D**). The overall leaf photosynthetic capacity, indicated by Rfd parameter, was elevated in *jac1* mutant in relation to the control plants (**Figure 6E**). Taken together, this ensemble of parameters shows JAC1 to negatively influence the photosynthetic activity under photooxidative stress.

**Figure 7:**
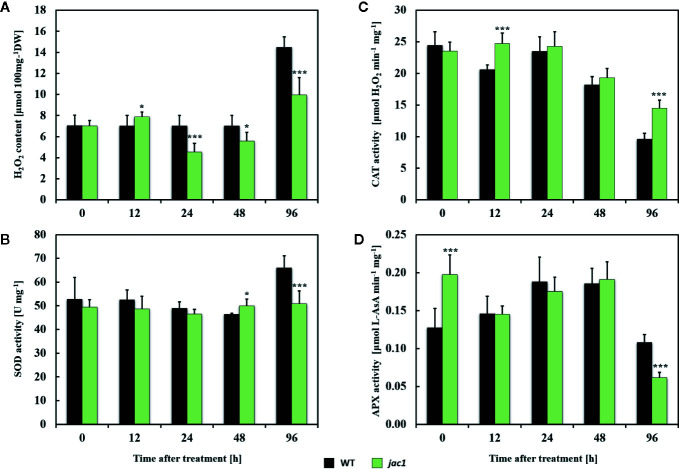
Hydrogen peroxide content and activities of selected antioxidant enzymes in 4-week-old *Arabidopsis thaliana* wild type and *jac1* mutant plants measured for untreated plants and 12, 24, 48, and 96 h after UV-C exposure (100 mJ cm^-2^). **(A)** H_2_O_2_, hydrogen peroxide content; **(B)** SOD, superoxide dismutase activity; **(C)** CAT, catalase activity; **(D)** APX, ascorbate peroxidase activity. Values are means (± SD) of 9 plants *per* genotype *per* time point from two independent experiments (n=9). Asterisks indicate significant difference in comparison with the wild-type plants at the level P<0.05 (*) or P<0.001 (***) according to the Tukey’s HSD test.

Next, to examine the role of JAC1 in UV-C-triggered photooxidative stress response, we analyzed the concentration of H_2_O_2_ and activities of antioxidant enzymes in *jac1* mutant and wild-type plants. Before the UV-C exposure, the H_2_O_2_ content, as well as superoxide dismutase (SOD) and catalase (CAT) activities were similar in *jac1* and wild-type plants (**Figures 7A–C**). In contrast, non-stressed *jac1* plants showed significantly higher activity of ascorbate peroxidase (APX) (**Figure 7D**).

Twelve hours after UV-C exposure, *jac1* mutant demonstrated higher levels of H_2_O_2_ (**Figure 7A**), which corresponded with elevated CAT activity (**Figure 7C**). In contrast, samples analyzed in all time points after 24 h *post* UV-C stress showed lower content of H_2_O_2_ (**Figure 7A**). The pattern of SOD and APX activities in *jac1* mutant was similar to the wild-type, except 96 h after UV-C exposure, when activities of those antioxidant enzymes were significantly decreased in relation to the controls (**Figures 7B, D**).

**Figure 8:**
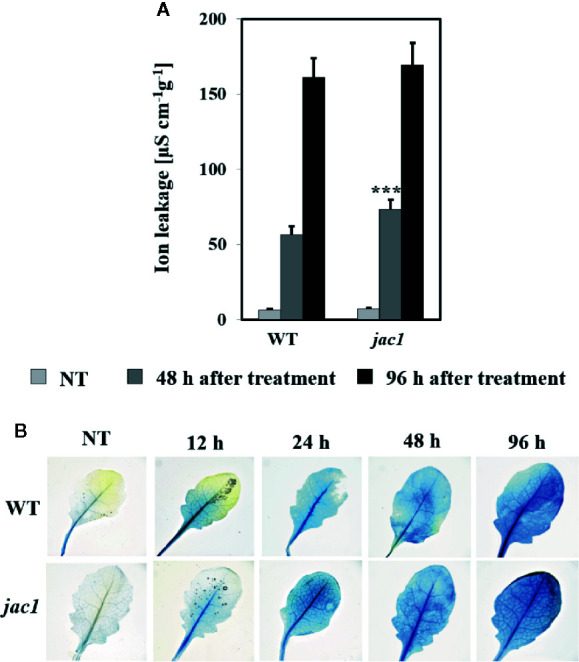
Cell death in 4-week-old *Arabidopsis thaliana* wild type and *jac1* mutant measured for untreated plants (NT) and 48 and 96 h after UV-C exposure (100 mJ cm^-2^). Cell death **(A)** quantified by cellular electrolyte leakage; and **(B)** visualized using Evans blue staining. Values are means (± SD) of 9–12 plants *per* genotype *per* time point from two independent experiments (n=9–12). Asterisks indicate significant difference in comparison with the wild-type plants at the level P < 0.001 (***) according to the Tukey’s HSD test.

In order to assess the influence of JAC1 activity on UV-C-triggered cell death, we performed the analysis of cellular leakage and Evans blue staining of dead cells. Our results proved that 48 h after UV-C treatment, *jac1* mutant demonstrated significantly higher ion leakage (**Figure 8A**). Higher cell death level was also confirmed by Evans blue staining (**Figure 8B**), which was visibly more intense at all time points starting from 12 h after UV-C stress.

### UV-C Stress Reshapes the Arabidopsis Transcriptome

In a next phase, we profiled gene expression changes under UV-C stress using high-throughput mRNA sequencing (RNA-seq). In the wild type, UV-C treatment resulted in 2,094 differentially expressed genes (DEGs) (FDR < 0.01, absolute log2 fold change (FC) > 1) (**Supplementary Data Sheet 2**). In total, 1,579 of these DEGs (75.4%) showed higher expression level after UV-C treatment. Gene set enrichment demonstrated that many DEGs encode membrane, cell wall and chloroplast proteins, especially those integral to thylakoids (**Supplementary Data Sheet 3**). Interestingly, 73 genes encoded by chloroplast genome were highly upregulated after UV-C treatment (**Supplementary Table 1**). In addition, amongst the DEGs, there were many genes encoding stress-responsive proteins, including transcription factors and proteins possessing oxidoreductase activity (**Supplementary Data Sheet 3**). There was also an enrichment of genes encoding tetrapyrrole (including chlorophyll)-binding proteins, light-harvesting complex proteins and photosystems subunits (**Supplementary Figure 5**), as well as biotic- and abiotic-stress responsive proteins (**Supplementary Figure 6**), mitochondrial electron transport chain proteins (**Supplementary Figure 7**), cell wall modification proteins, proteins involved in hormone and redox homeostasis, and protein modifying/degrading enzymes (**Supplementary Figure 8**). These results indicate that UV-C treatment in wild-type plants has pronounced effects on gene expression level.

### Changes in *jac1* Transcriptome in Non-Stress and After Oxidative Stress Conditions

Under control conditions, 558 DEGs were apparent in *jac1* in comparison to the wild type (FDR < 0.01, |log2 FC| > 1) (**Supplementary Data Sheet 2**). Enrichment analysis showed that relatively high proportion of DEGs encoded extracellular (16%) and cell wall (6%) proteins (**Supplementary Data Sheet 4**). In terms of biological processes, *jac1* mutant, in relation to the wild type, demonstrated changes in expression level of genes encoding proteins involved in abiotic stresses (mainly light-induced stress), circadian rhythm and transcriptional regulation (**Supplementary Data Sheet 4**). Given the observed redox- and photosynthetic-related changes in non-stressed *jac1*, we inspected the gene expression of several photosynthetic components and redox homeostasis enzymes. We observed induced expression of both LHCI (LHCA1, LHCA4) and LHCII (LHCB1.1, LHCB1.2, LHCB1.4, LHCB2.1, LHCB2.2, LHCB2.3, LHCB3) components in *jac1*, in comparison to the wild type (**Supplementary Data Sheet 4**). Furthermore, in non-stress conditions we found five DEGs involved in redox regulation, encoding thioredoxins, glutaredoxins, and enzymes involved in ascorbate and glutathione metabolism. For instance, monodehydroascorbate reductase (ATMDAR, AT3G09940) was highly induced in *jac1* background. This enzyme takes part in ascorbate recovery, which provides reducing power to APX. In addition, we observed a relatively high number of DEGs encoding cell wall modifying enzymes in non-treated *jac1*, compared to the wild type. Also, genes encoding proteins involved in proteolysis and signalling, especially light- and calcium-induced signal transduction demonstrated changed expression in *jac1*, in relation to wild-type plants. Interestingly, seven genes involved in calcium signalling expressed in stomata guard cells were significantly induced in *jac1* when compared to the wild type (**Supplementary Data Sheet 4**): *MULTICOPY SUPPRESSORS OF SNF4 DEFICIENCY IN YEAST 3* (AT2G43290), calmodulin binding *SAR DEFICIENT 1* (AT1G73805), *CALMODULIN LIKE 23* (AT1G66400), *CALMODULIN 9* (AT3G51920), *TOUCH 2* (AT5G37770), *TOUCH 3* (AT2G41100) and a calcium-binding endonuclease/exonuclease/phosphatase family gene (AT5G54130) (Obulareddy et al., 2013). Moreover, a gene encoding calcium-binding EF hand family protein (AT4G27280), engaged in stomata movements (Cominelli et al., 2005) was up-regulated in *jac1*, compared to the wild type. CHLORIDE CHANNEL B (AT3G27170), and two water channels, PLASMA MEMBRANE INTRINSIC PROTEIN 2 (AT3G53420) and RESPONSIVE TO DESICCATION 28 (AT2G37180), all expressed in guard cells (Obulareddy et al., 2013; Wang et al., 2016), also demonstrated significantly higher expression level in *jac1* mutant, compared to wild-type counterparts.

After UV-C stress, *jac1* displayed 1,017 DEGs in comparison to wild-type plants (**Supplementary Data Sheet 2**). Within the “cellular component” category we observed enrichment in genes encoding components of cell wall, plasma membrane and photosystems (**Supplementary Data Sheet 5**). DEGs in UV-treated *jac1* *versus* UV-treated wild type were over-represented by genes encoding proteins engaged in response to stimulus (especially abiotic stress), and response to hormones. Among stress-related genes, we identified genes encoding proteins involved in oxidative stress signalling and response, such as catalase 3 (CAT3, AT1G20620), galactinol synthase 2 (GolS2, AT1G56600), and GolS3 (AT1G09350) to be up-regulated, in comparison to the wild type (Sun et al., 2013; Song et al., 2016; Selvaraj et al., 2017). Moreover, significantly changed expression level in UV-C treated *jac1* background, in relation to the wild type, included genes encoding transcription factors (10% of all the DEGs), enzymes with oxidoreductase activity (8% of all the DEGs), tetrapyrole-binding proteins (3% of all the DEGs). Furthermore, MapMan categorization proved that after UV-C stress in *jac1* mutant, compared to wild-type plants, there were a lot of genes (both up- and down-regulated) encoding proteins involved in signal transduction (i.e. receptor kinases, proteins involved in light-, calcium-dependent signalling), intra- and inter-cellular transport, protein degradation and development (i.e. senescence-associated genes) (**Supplementary Data Sheet 5**). After UV-C treatment, similarly to non-treated samples, *jac1* demonstrated higher expression level of LHCI (LHCA1, LHCA3, LHCA4) and LHCII (LHCB1.1, LHCB1.2, LHCB1.4, LHCB2.2, LHCB2.4, LHCB3, LHCB4.2) components.

## Discussion

Under variable natural conditions plants need to optimize the absorption of light in order to efficiently perform photosynthetic reactions. EEE, which is the energy absorbed in excess, may lead to photoinhibition, photosystem damage, chloroplasts disruption, and finally to cell death. Thus, plants are equipped in various avoidance and dissipation mechanisms that enable them to protect photosynthetic apparatus from EEE and perform optimal, condition-dependent photosynthesis.

Among the EEE protective mechanisms we can distinguish chloroplast movements, NPQ, and state-transitions (Karpiński et al., 2013; Gawroński et al., 2014; Gilroy et al., 2016; Czarnocka and Karpiński, 2018; Górecka et al., 2020). Chloroplast accumulation and avoidance response are dependent on blue light and UV-A/B radiation receptors, phototropins, and a non-receptor protein, JAC1 (Suetsugu et al., 2005; Kodama et al., 2010; Ichikawa et al., 2011). Even though the JAC1 function was well described in terms of chloroplast movements, so far little was known about its involvement in photosynthetic reactions, response to EEE and cell death.

The analysis of chlorophyll *a* fluorescence performed in this study proved that *jac1* mutant has elevated value of maximum quantum efficiency of PSII (*F_v_/F_m_*), more active centres, better connectivity between the structurally heterogeneous PSII units and lower Q_A_ pool reduction, compared to the wild type. The results indicate that even though the mutation in JAC1 positively influences PSII maximum quantum efficiency (*F_v_/F_m_*), it has negative impact on the conversion of the absorbed photon energy into photochemistry (*qP*). Higher *F_v_/F_m_* in *jac1*, compared to the wild type, may be connected with the lack of chloroplast accumulation ability and thus the need of induced potential photosynthetic efficiency and suggests that the *jac1* mutant has elevated capacity of acclimation to variable light conditions, in relation to the wild type. Moreover, higher *F_v_/F_m_* in *jac1* *versus* wild type was in agreement with elevated level of total chlorophyll and up-regulation of genes encoding LHC subunits.

We also observed decreased values of NPQ in *jac1* mutant, compared to the wild type, which well corresponded with decreased xanthophyll pigments level. Xanthophyll pigments are involved in light energy dissipation during violaxanthin-antheraxanthin-zeaxanthin (VAZ) cycle (Demmig-Adams and Adams, 1992) and thus protect plants against photodamage (Havaux and Niyogi, 1999). Lower NPQ value and reduced content of xanthophyll pigments in *jac1*, in relation to the wild type, may be caused by the defects in *jac1* chloroplasts photorelocation. Because the *jac1* chloroplasts are constantly located close to the anticlinal cell walls, low light-acclimated mutant has less necessity in energy quenching. These results indicate that JAC1-dependent chloroplast movement positively influences non-photochemical reaction rates and light energy dissipation.

Most importantly, we proved that apart from positively affecting photosynthetic electron transport, JAC1 negatively influences plant CO_2_ uptake. The *jac1* mutant demonstrated significantly elevated CO_2_ uptake rate, compared to the wild type, especially in higher light intensities. This observation was most probably caused by the chloroplasts inhibited mobility. It was shown that chloroplast surface area exposed to intercellular airspaces positively correlates with the CO_2_ conductance, and thus it was suggested that chloroplast movement control the CO_2_ uptake by modulating the CO_2_ diffusion path length from intercellular airspaces to the chloroplast stroma (Terashima et al., 2006). It was also proven that the chloroplast avoidance response decreases the internal conductance to CO_2_ diffusion in Arabidopsis leaves (Tholen et al., 2008). Taking into consideration that *jac1* mutant is to some extent disturbed in avoidance response (Kodama et al., 2010; Ichikawa et al., 2011), relatively higher chloroplast surface area should be adjacent to intercellular airspaces, in comparison to wild-type plants, especially in high light intensities. Therefore, the diffusion path length of CO_2_ to the chloroplast stroma may be lower in *jac1*, in relation to control plants, which can be one of the factors increasing the CO_2_ uptake rate in *jac1* mutant. Furthermore, higher variation of CO_2_ uptake and GH_2_O parameters in a single measuring point in *jac1* mutant, compared to the wild type, suggests that the lack of functional JAC1 affects the ability of whole-leaf stomatal aperture stabilization, most likely due to changes in the guard cells functioning.

Increased CO_2_ uptake rate, correlated with JAC1 absence, was connected with almost three-times elevated water vapor conductance and two-times higher stomatal aperture. Performed transcriptomic profiling showed that many genes, expressed in stomata guard cells and involved in stomata movements, were all up-regulated in *jac1*, compared to wild-type counterparts. These were genes encoding proteins involved in calcium binding (Cominelli et al., 2005; Obulareddy et al., 2013) as well as chloride and water channel proteins (Obulareddy et al., 2013; Wang et al., 2016). Therefore, it indicates that JAC1 activity is genetically coupled with the negative regulation of stomatal conductance, and thus, affects water vapor conductance and CO_2_ uptake rate. We cannot exclude that JAC1 regulates the gene expression directly, because of its possible presence in the nucleus (Suetsugu et al., 2005). Higher stomatal aperture and water vapor conductance in *jac1* mutant were most probably the reason for higher transpiration and water loss. Therefore, JAC1 activity positively influenced water use efficiency (WUE). Despite these changes, we did not observe any statistically important changes in *jac1* mutant size or dry weight, as compared to the wild-type plants. Interestingly, elevated CO_2_ uptake in *jac1* mutant did not correlate with increased biomass production. Our observations are contrary to the study by Gotoh and co-workers (2018), where reduced photosynthesis, CO_2_ uptake, smaller rosette size and lesser biomass production were observed for *jac1* mutant. However, this research was performed in moderate light conditions (120 µmol photons m^-^² s^-^¹), while the experiments described in the current work were all performed for relatively low light conditions (80 µmol photons m^-^² s^-^¹). This may indicate that, similarly to our previous studies on the regulators of EEE responses, such as LSD1 (Wituszyńska et al., 2013b; Wituszyńska et al., 2015), the role of JAC1 may be condition-dependent. Therefore, we postulate that JAC1 may participate in the condition-dependent optimization of the photon energy use in photosynthetic light phase reaction and the CO_2_ uptake.

We have shown before that UV-C treatment induces chloroplast retrograde signalling, causes photoinhibition, photooxidative stress and finally cell death (Rusaczonek et al., 2015; Wituszyńska et al., 2015). Since chloroplast movement is an important mechanism of EEE avoidance, we wanted to define the role of JAC1 protein in the activation of UV-C triggered cellular response. In a very recent study, it was shown that UV-B treatment did not trigger chloroplast rearrangements in the upper parts of palisade cells in the *jac1* mutant, which means that upon UV-B, the *jac1* chloroplasts were stuck and did not demonstrate avoidance response (Hermanowicz et al., 2019). Despite the greater exposure of chloroplasts to UV-C, we demonstrated that the *jac1* mutant had higher values of maximum and operational quantum efficiency of PSII, and the overall leaf photosynthetic capacity (Rfd), when compared to the wild-type counterparts, thus indicating that JAC1 activity negatively affects the PSII protection under UV-C stress. These results suggest that JAC1 influences acclimatory processes, leading to LHCs and PS photo-protection.

When assessing differences in H_2_O_2_ levels and antioxidant enzyme activities in *jac1* mutant and wild type plants after UV-C exposure, *jac1* mutant showed higher level of H_2_O_2_ 12 h after treatment. In contrast, in later time points, H_2_O_2_ levels were decreased, when compared to the wild type. The H_2_O_2_ content 12 h after UV-C exposure corresponded well with the CAT activity, which was higher in *jac1*, when compared to the wild type, and thus processed H_2_O_2_ more efficiently. In fact, transcriptomic profiling proved that the expression of CAT3 (AT1G20620), located in peroxisomes (Li et al., 2015), was up-regulated in *jac1* background after UV-C treatment. Additionally, in non-treated *jac1* we observed higher APX activity, which corresponded with higher expression of monodehydroascorbate reductase (ATMDAR), engaged in ascorbate recovery.

Moreover, UV-induced cell death was more pronounced in *jac1* mutant after 48 h, but not after 96 h post UV-C exposure, which suggests that JAC1 negatively influences the fine-tuning of cell death signalling and progression. The only work so far studying the impact of JAC1 on stress response showed that Arabidopsis line with significantly higher expression of JAC1 demonstrated lower level of aluminium-induced oxidative stress and higher resistance towards Al stress, than the wild-type plants (Ezaki et al., 2007). This study is convergent with our conclusion on the role of JAC1 in the increasing of resistance towards cell death. Interestingly, the expression level of JAC1 demonstrated dynamic pattern, as it was up-regulated 3 and 6 h after UV-C exposure and strongly down-regulated afterwards. It seems that Arabidopsis wild-type plants tend to diminish JAC1 expression to reduce its negative impact on photosynthetic activity and lower the spread of cell death under oxidative stress. All these results indicate that JAC1 is most important at the early stages of photooxidative stress as it is engaged in the fine-tuning of the cellular redox homeostasis and cell death.

In the present work, we also identified genes that are transcriptionally affected by the absence of JAC1 activity. Interestingly, in *jac1* mutant, when compared to the wild type, we found deregulation of many genes encoding proteins engaged in calcium signalling, such as calmodulins (CaMs), CaM-like and CaM-binding proteins. It was previously shown that JAC1 binds calmodulin 1 (CaM1) and CaM-like CML10 (Popescu et al., 2007). Here, we prove that the regulation of CaMs is not only at the protein-protein interaction level, but also at the transcriptional stage. Importantly, two times more genes showed significant change in the expression level in UV-C treated *jac1*, compared to the UV-C treated wild type. It may be caused by altered chloroplast positioning in *jac1*, that due to impaired avoidance response capture more UV-C and hence provoke more plastid signals affecting nuclear gene expression. Although we have identified many genes that are influenced by the lack of JAC1 activity, the identification of JAC1-interacting proteins may further clarify the role of JAC1 in chloroplast movement, photosynthetic reactions, stomatal conductance, and stress responses.

In conclusion, we have identified a new role of JAC1 in the photosynthetic apparatus efficiency and plant stomatal conductance and thus CO_2_ uptake. Our results prove that the movements of stomata are genetically coupled with JAC1 activity. From a broader perspective, we demonstrate that chloroplast altered positioning, dependent on JAC1, influences plant photosynthetic performance, H_2_O_2_ foliar levels, antioxidant enzymes activities, and cell death after UV-C photooxidative stress.

## Data Availability Statement

The datasets presented in this study can be found in online repositories. The names of the repository/repositories and accession number(s) can be found in the article/**Supplementary Material**.

## Author Contributions

WC, AR, and SK planned the experiments and postulated the hypotheses tested in this paper. WC measured hydrogen peroxide content and activities of antioxidant enzymes, prepared the RNA for NGS sequencing, performed the qPCRs, and analyzed functionally the RNAseq results. AR performed morphological analyses (measured plant size, dry weight, stomatal density), analyzed water use efficiency, chlorophyll *a* fluorescence, and ion leakage. PW did the annotation and statistical analysis of the RNAseq data. MS-R performed analysis of stomatal aperture and Evans Blue staining. WC wrote the manuscript and together with AR prepared the figures. SK and FB reviewed and approved its final version.

## Conflict of Interest

The authors declare that the research was conducted in the absence of any commercial or financial relationships that could be construed as a potential conflict of interest.
