# Supplementary material for: Novel Role of JAC1 in Influencing Photosynthesis, Stomatal Conductance, and Photooxidative Stress Signalling Pathway in Arabidopsis thaliana
Source: Front Plant Sci. 2020 Jul 29;11:1124. doi: 10.3389/fpls.2020.01124 (PMC7403226; doi:10.3389/fpls.2020.01124)
Supplement: Supplementary file 1 [file DataSheet_1.pdf]

Supplementary material

Novel role of JAC1 in influencing photosynthesis, stomatal conductance and photooxidative stress signalling pathway in *Arabidopsis thaliana*

Czarnocka Weronika [1,2]*, Rusaczonek Anna [1,2], Willems Patrick [3,4], Sujkowska-Rybkowska Marzena [1], Van Breusegem Frank [3,4], Karpiński Stanisław [2]

[1] Department of Botany, Institute of Biology, Warsaw University of Life Sciences, Nowoursynowska 159, 02-776 Warsaw, Poland
[2] Department of Plant Genetics, Breeding and Biotechnology, Institute of Biology, Warsaw University of Life Sciences, Nowoursynowska 159, 02-776 Warsaw, Poland
[3] Department of Plant Biotechnology and Bioinformatics, Ghent University, Technologiepark 71, 9052 Ghent, Belgium
[4] Center of Plant Systems Biology, VIB, Technologiepark 71, 9052 Ghent, Belgium

*** Correspondence:**
Weronika Czarnocka
weronika_czarnocka@sggw.edu.pl

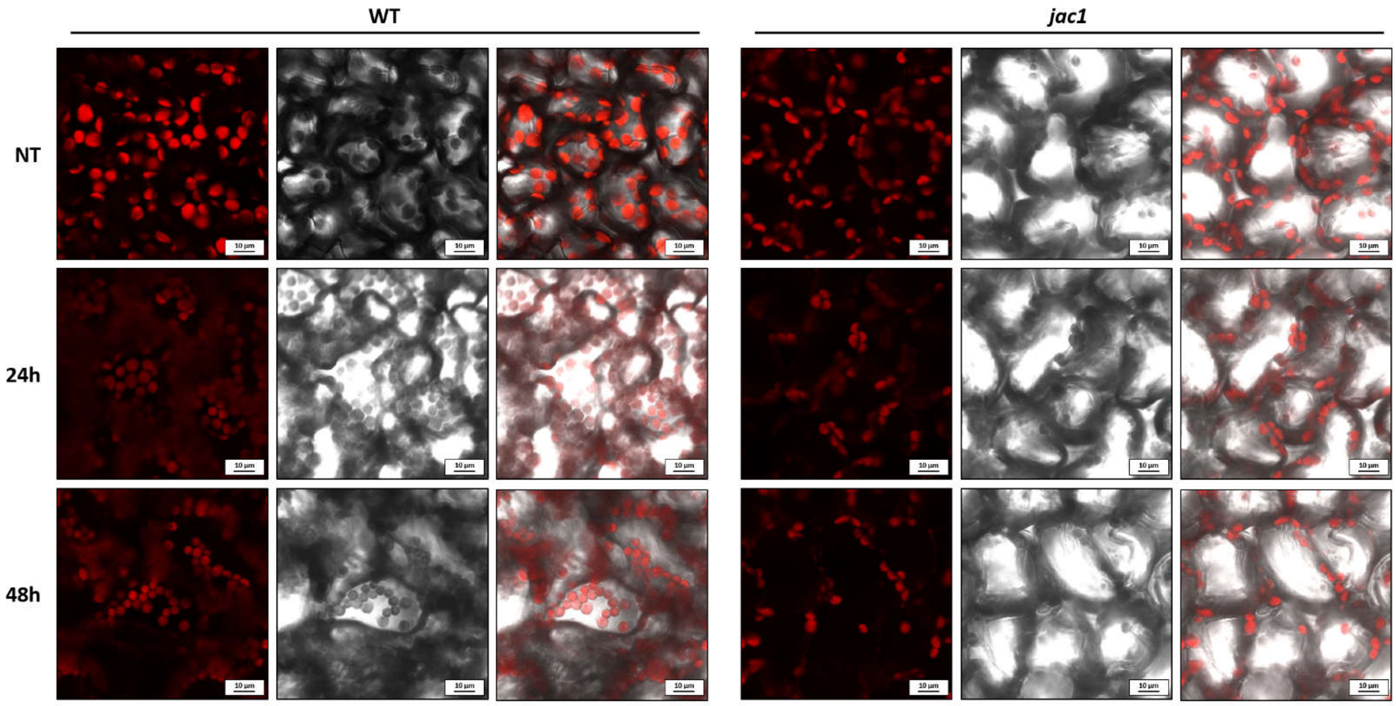

Supplementary Figure 1. Intracellular chloroplast location in leaf mesophyll cells of *Arabidopsis thaliana* wild type (WT) and *jac1* mutant, in non-stress (NT) conditions and 24 h and 48 h after UV-C treatment. Chloroplasts were imaged with a confocal microscope, using chlorophyll autofluorescence (in red). Scale bar = 10 µm.

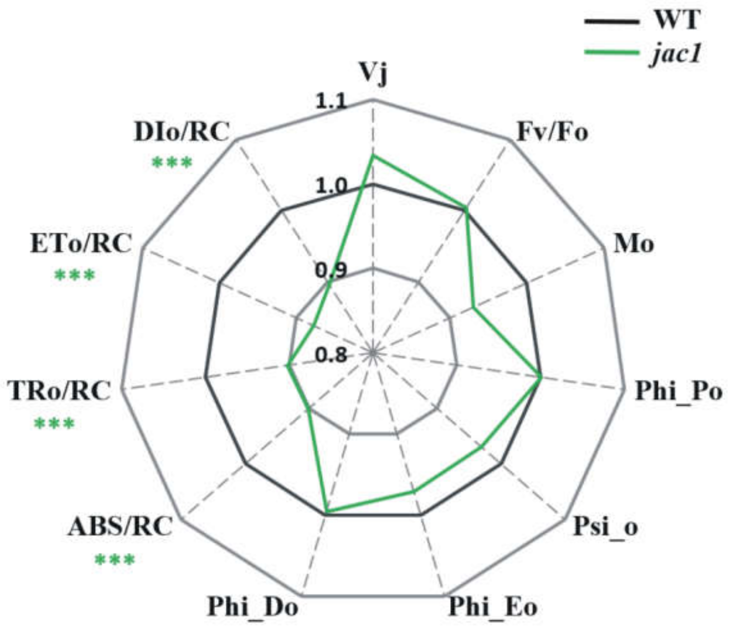

Supplementary Figure 2. Fluorescence parameters from the OJIP test for *Arabidopsis thaliana* wild type (WT) and *jac1* mutant. Vj, relative variable fluorescence at the J-step; *Fv/Fo*, efficiency of the oxygen-evolving complex; Mo, approximated initial slope of the fluorescence transient; Phi_Po, maximum quantum yield of primary photochemistry; Psi_o, probability that a trapped exciton moves an electron into the electron transport chain beyond Q_{A-}; Phi_Eo, quantum yield of electron transport; Phi_Do, quantum yield of energy dissipation; ABS/RC, absorption flux per reaction centre; TRo/RC, trapped energy flux per reaction centre; ETo/RC, electron transport flux per reaction centre; DIo/RC, dissipated energy flux per reaction centre. Values are means (±SD) of 11-21 plants per genotype from two independent experiments (n=11-21). Asterisks indicate significant difference in comparison with the wild-type plants at the level P<0.001 (***), according to the Tukey's HSD test.

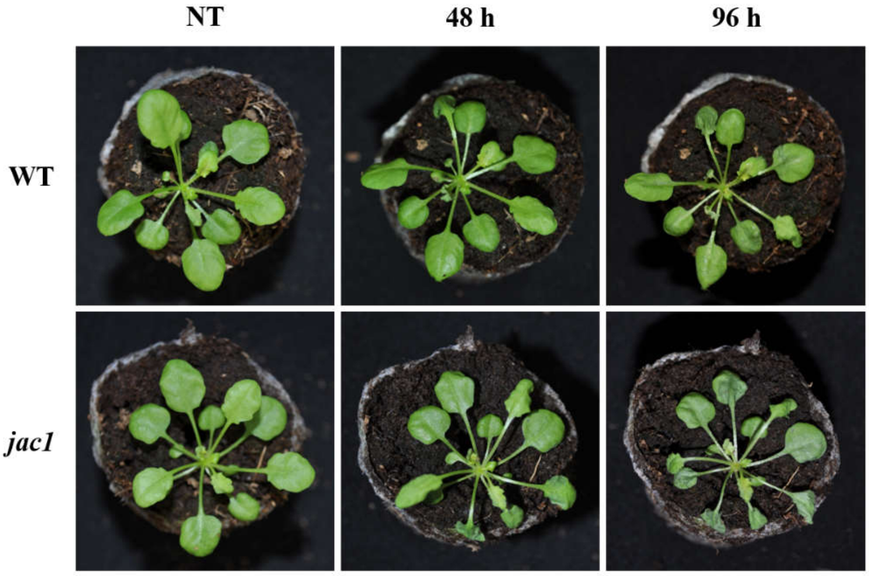

Supplementary Figure 3. Rosette morphology of *Arabidopsis thaliana* wild type (WT) and *jac1* mutant before, 48 and 96 h after UV-C exposure (100 mJ cm^{-2}).

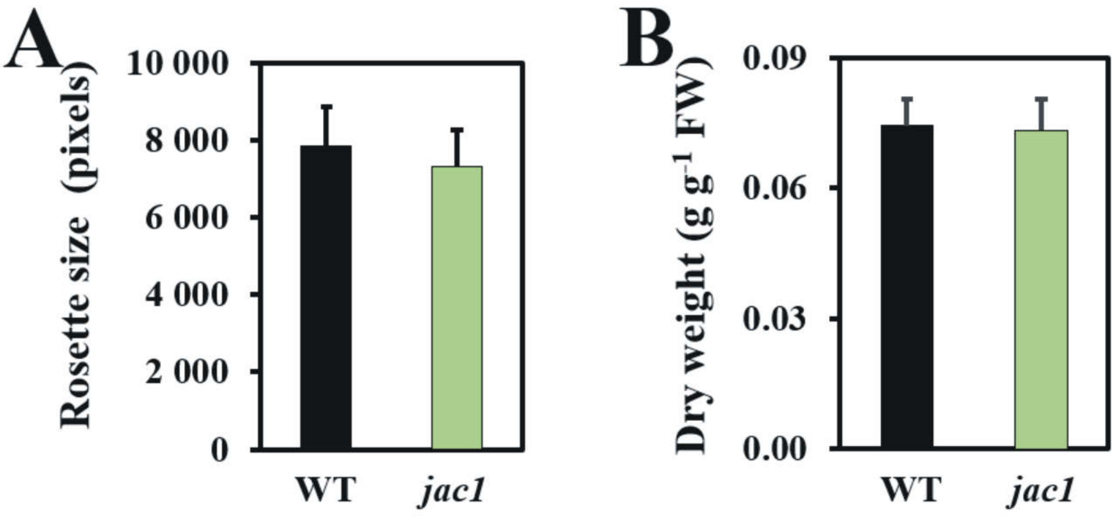

Supplementary Figure 4. Morphological traits of 4-week-old *Arabidopsis thaliana* wild type (WT) and *jac1*. (A) Rosette size. Values are means (±SD) of 15 plants per genotype from two independent experiments (n=15); (B) Dry weight. Values are means (±SD) of 12 plants per genotype from at least two independent experiments (n=12). The statistical analysis was performed using the Tukey's HSD test.

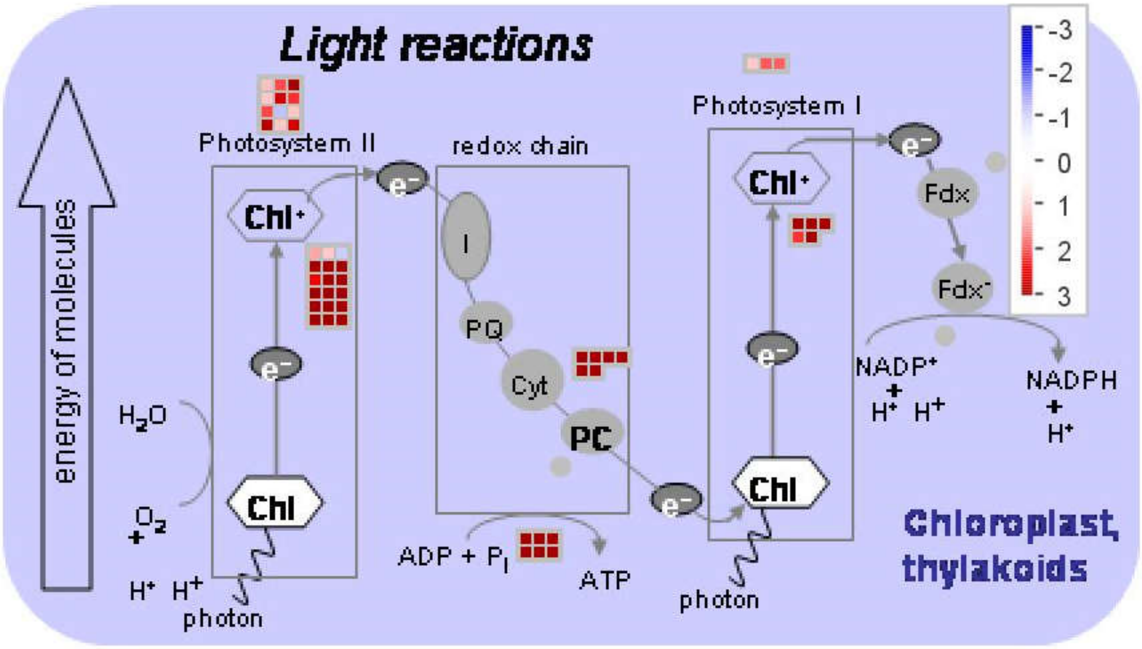

Supplementary Figure 5. Graphical representation of deregulated photosynthesis-related genes in UV-C treated wild type plants *versus* non-treated.

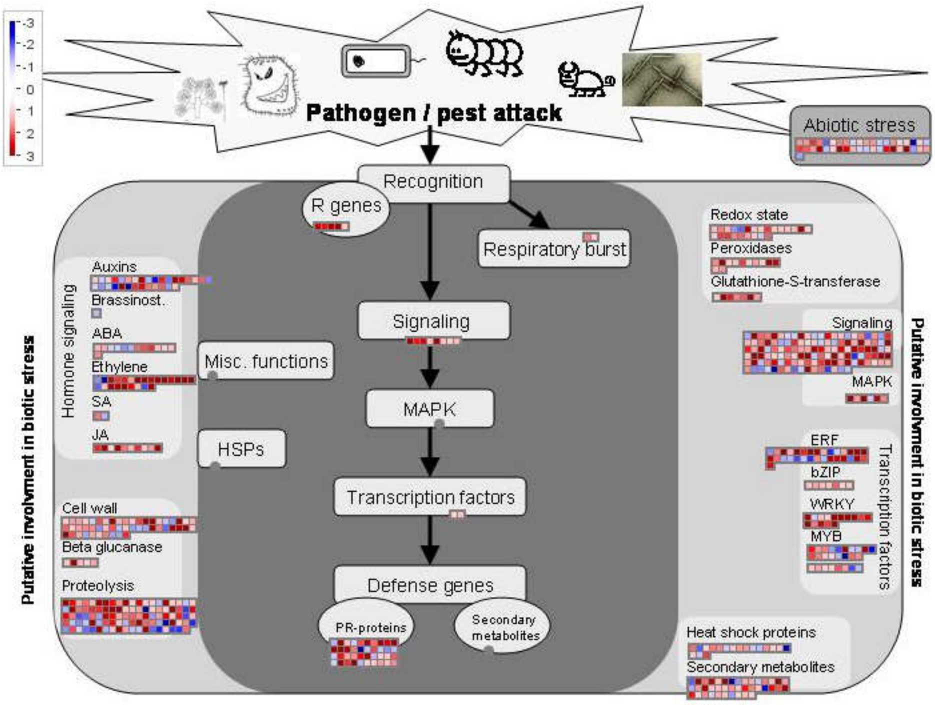

Supplementary Figure 6. Graphical representation of deregulated stress-related genes in UV-C treated wild type plants *versus* non-treated.

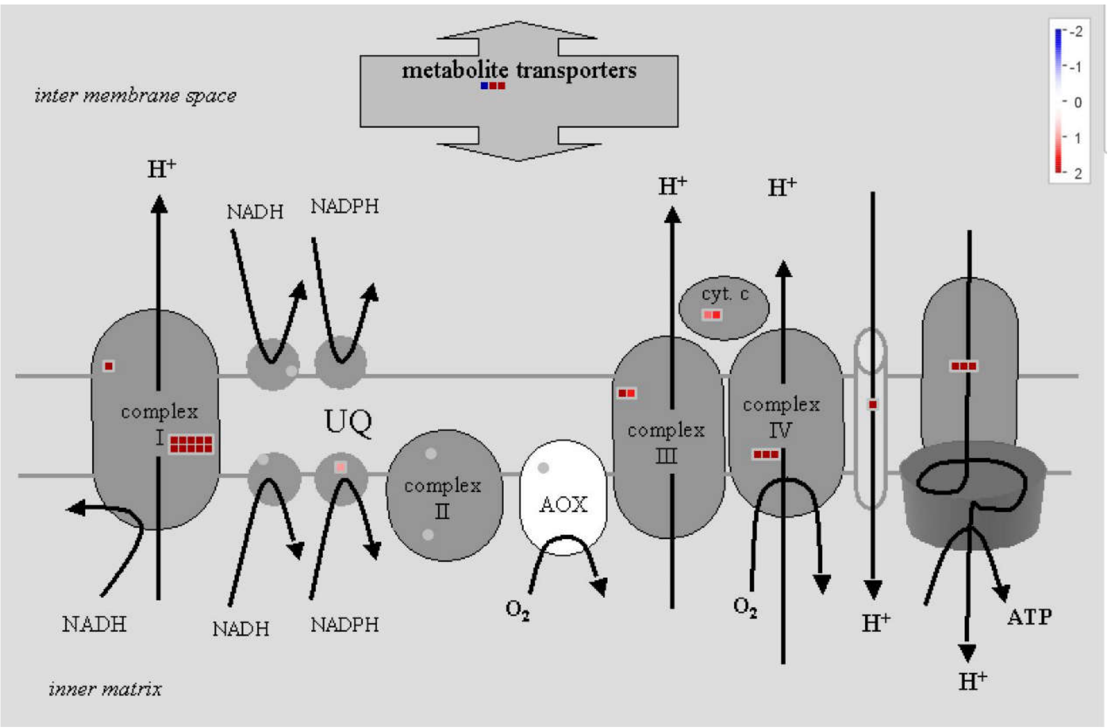

Supplementary Figure 7. Graphical representation of deregulated genes related to mitochondrial electron transport chain in UV-C treated wild type plants *versus* non-treated.

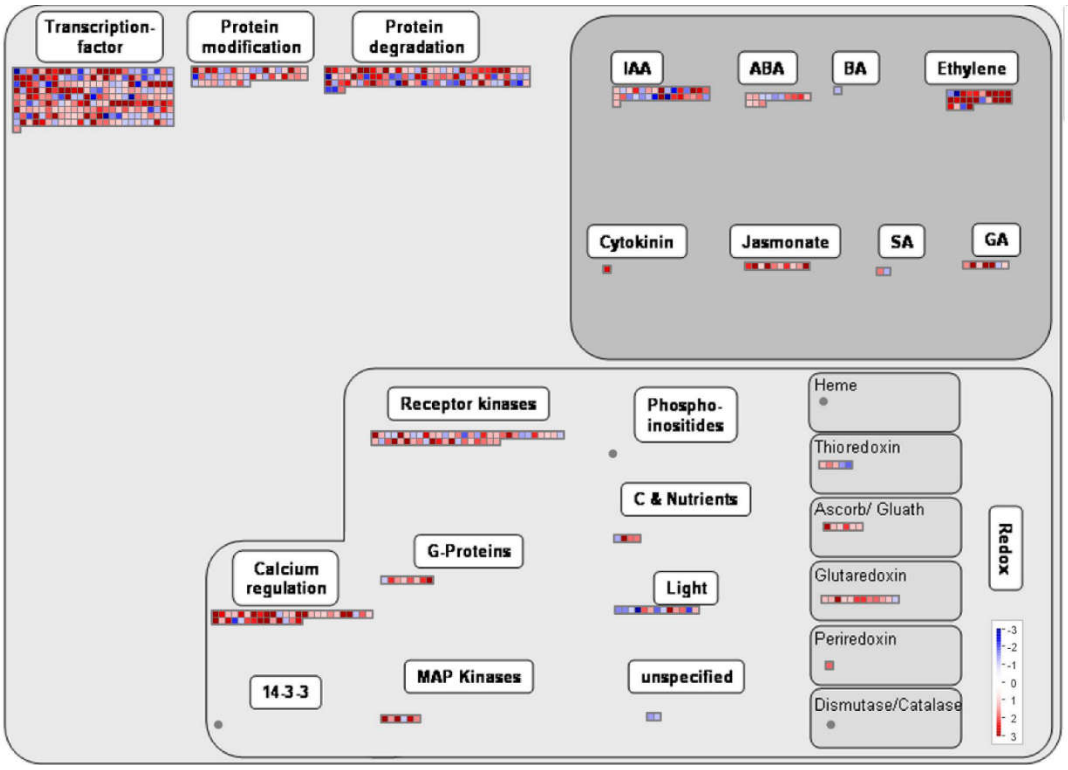

Supplementary Figure 8. Graphical representation of deregulated genes related to transcriptional regulation, protein modification/degradation, hormonal and redox regulation, in UV-C treated wild type plants *versus* non-treated.

Supplementary Table 1. Chloroplast genome encoded genes.

Gene	Description	WT: UV vs. NT (log2FC)	jac1: UV vs. NT (log2FC)	Non-treated: jac1 vs WT (log2FC)	UV: jac1 vs. WT (log2FC)
ATCG00490	ribulose-bisphosphate carboxylases	8,691	9,073	-0,160	0,222
ATCG00350	Photosystem I, PsaA/PsaB protein	8,266	8,290	0,245	0,269
ATCG00520	unfolded protein binding	8,120	7,534	0,545	-0,041
ATCG00020	photosystem II reaction center protein A	7,895	7,925	-0,033	-0,003
ATCG00280	photosystem II reaction center protein C	7,852	8,089	0,029	0,266
ATCG00270	photosystem II reaction center protein D	7,802	7,608	0,364	0,170
ATCG01110	NAD(P)H dehydrogenase subunit H	7,768	7,817	0,080	0,130
ATCG00720	photosynthetic electron transfer B	7,740	7,720	0,271	0,251
ATCG01100	NADH dehydrogenase family protein	7,739	7,789	0,132	0,182
ATCG00710	photosystem II reaction center protein H	7,697	7,821	0,178	0,301
ATCG00040	maturase K	7,616	7,908	-0,262	0,030
ATCG01080	NADH:ubiquinone/plastoquinone oxidoreductase, chain 6	7,582	7,321	0,517	0,256
ATCG01090	NADPH dehydrogenases	7,578	8,645	-0,905	0,162
ATCG00700	photosystem II reaction center protein N	7,522	7,964	-0,203	0,239
ATCG00580	photosystem II reaction center protein E	7,433	7,428	0,140	0,135
ATCG01070	NADH-ubiquinone/plastoquinone oxidoreductase chain 4L	7,381	7,486	-0,250	-0,144
ATCG00340	Photosystem I, PsaA/PsaB protein	7,348	7,323	0,274	0,249
ATCG00050	ribosomal protein S16	7,292	8,182	-1,537	-0,647

ATCG00680	photosystem II reaction center protein B	7,287	7,404	0,138	0,255
ATCG01060	iron-sulfur cluster binding;electron carriers	7,239	7,532	0,060	0,353
ATCG00570	photosystem II reaction center protein F	7,112	7,412	-0,153	0,147
ATCG00500	acetyl-CoA carboxylase carboxyl transferase subunit beta	7,088	7,569	-0,285	0,196
ATCG00300	YCF9	7,007	10,339	-3,837	-0,506
ATCG00730	photosynthetic electron transfer D	6,993	6,832	0,404	0,244
ATCG00210	electron transporter, transferring electrons within PSII	6,793	5,928	0,816	-0,049
ATCG00130	ATPase, F0 complex, subunit B/B', bacterial/chloroplast	6,785	6,891	0,298	0,404
ATCG00140	ATP synthase subunit C family protein	6,651	6,846	0,288	0,483
ATCG01120	chloroplast ribosomal protein S15	6,616	6,419	0,112	-0,085
ATCG01010	NADH-Ubiquinone oxidoreductase (complex I), chain 5 protein	6,560	7,354	-0,779	0,014
ATCG00330	chloroplast ribosomal protein S14	6,548	6,697	0,109	0,258
ATCG00650	ribosomal protein S18	6,530	6,469	0,183	0,122
ATCG00820	ribosomal protein S19	6,468	6,575	0,123	0,230
ATCG00560	photosystem II reaction center protein L	6,410	5,816	0,288	-0,306
ATCG00540	photosynthetic electron transfer A	6,387	6,166	0,364	0,142
ATCG00440	NADH:ubiquinone/plastoquinone oxidoreductase, chain 3	6,372	5,823	0,683	0,134
ATCG00360	Tetratricopeptide repeat (TPR)-like superfamily protein	6,368	5,868	0,640	0,141
ATCG00120	ATP synthase subunit alpha	6,305	6,284	0,357	0,336
ATCG00150	ATPase, F0 complex, subunit A protein	6,255	6,248	0,242	0,235
ATCG00640	ribosomal protein L33	6,102	5,517	0,570	-0,015
ATCG00430	photosystem II reaction center protein G	5,986	6,181	0,128	0,322
ATCG00380	chloroplast ribosomal protein S4	5,966	5,588	0,504	0,126
ATCG00420	NADH dehydrogenase subunit J	5,907	5,890	0,263	0,246
ATCG01040	Cytochrome C assembly protein	5,712	5,570	0,246	0,104
ATCG00530	CemA-like proton extrusion protein-related	5,684	4,640	0,740	-0,304
ATCG00600	PETG	5,632	6,388	-1,099	-0,343
ATCG00690	photosystem II reaction center protein T	5,537	5,918	-0,210	0,171
ATCG01050	NADH-Ubiquinone/plastoquinone (complex I) protein	5,433	5,820	-0,498	-0,111
ATCG00550	photosystem II reaction center protein J	5,308	5,308	0,116	0,116
ATCG00750	ribosomal protein S11	5,289	4,987	0,594	0,293
ATCG00065	ribosomal protein S12A	5,277	5,431	0,024	0,178
ATCG00660	ribosomal protein L20	5,252	6,040	-0,329	0,459
ATCG01130	Ycf1 protein	5,111	4,692	0,174	-0,246
ATCG00160	ribosomal protein S2	5,077	5,267	0,025	0,215
ATCG00510	photsystem I subunit I	4,901	6,567	-1,752	-0,086
ATCG00760	ribosomal protein L36	4,762	4,304	0,625	0,168
ATCG00770	ribosomal protein S8	4,759	4,105	0,651	-0,003
ATCG00170	DNA-directed RNA polymerase family protein	4,739	4,240	0,613	0,113
ATCG00740	RNA polymerase subunit alpha	4,734	4,472	0,289	0,027
ATCG00590	electron carriers	4,617	4,826	-0,620	-0,411
ATCG00800	structural constituent of ribosome	4,417	3,748	0,651	-0,018
ATCG00480	ATP synthase subunit beta	4,294	4,166	0,428	0,301
ATCG00810	ribosomal protein L22	4,183	3,786	0,539	0,143
ATCG00470	ATP synthase epsilon chain	4,181	3,890	0,520	0,229
ATCG00180	DNA-directed RNA polymerase family protein	3,890	3,480	0,526	0,116
ATCG00780	ribosomal protein L14	3,858	3,787	0,310	0,239
ATCG00070	photosystem II reaction center protein K precursor	3,827	3,575	0,277	0,024
ATCG00670	plastid-encoded CLP P	3,710	3,525	0,421	0,236
ATCG00790	ribosomal protein L16	3,677	3,521	0,564	0,407
ATCG00080	photosystem II reaction center protein I	3,148	3,151	-0,048	-0,045
ATCG00220	photosystem II reaction center protein M	2,458	1,757	0,278	-0,423
ATCG00190	RNA polymerase subunit beta	2,375	2,052	0,537	0,213
ATCG00630	PSAJ	2,153	1,613	0,178	-0,363
ATCG01020	ribosomal protein L32	1,611	1,146	0,181	-0,284
